# Interpretation of diffuse scattering in the high-*T*_c_ superconductor HgBa_2_CuO_4+δ_

**DOI:** 10.1107/S2052252516010629

**Published:** 2016-07-13

**Authors:** T. R. Welberry, D. J. Goossens

**Affiliations:** a Research School of Chemistry, Australian National University, Canberra, ACT 2601, Australia; b School of Physical, Environmental and Mathematical Sciences, University of New South Wales, Canberra, ACT 2600, Australia

**Keywords:** high-*T*_c_ superconductors, HgBa_2_CuO_4+δ_, diffuse X-ray scattering, improved model

## Abstract

Published diffuse X-ray scattering from the high-*T*_c_ superconductor HgBa_2_CuO_4+δ_ has been reexamined with a view to developing a model that more satisfactorily accounts for the observed patterns. The present work agrees with the previous conclusion that the doping oxygen atoms form long and isolated interstitial chains that run in both tetragonal directions. However, a distinctly different model is proposed for the accompanying displacement patterns of the atoms surrounding these linear defects. In this new model it is proposed that it is the correlated shifts of the Ba atoms along the length of the defect chains that are the primary source of the observed scattering, and that the variations of intensity in the generated diffuse streaks of scattering originate from lateral shifts of both Hg and Ba atoms away from defects. The new model yields diffraction patterns that are in much more convincing agreement with the observations than the original model.

## Abstract 2

Published diffuse X-ray scattering from the high-*T*_c_ superconductor HgBa_2_CuO_4+δ_ has been re-examined with a view to developing a model that more satisfactorily accounts for the observed patterns. It is proposed that it is correlated shifts of the Ba atoms along the length of the defect chains that are the primary source of the observed diffuse scattering lines and that the variations of intensity within these lines originate from lateral shifts of both Hg and Ba atoms away from the defects.

## 1. Introduction   

The properties of many technologically important materials are intimately associated with the inherent disorder that exists in their crystal structures. There are numerous examples in fields ranging from alloys (Schweika, 1998 ▸), shape-memory alloys (Yamamoto *et al.*, 2008 ▸), ferroelectrics (Pasciak *et al.*, 2012 ▸), fast ion conductors (Welberry & Pasciak, 2011 ▸; Keen, 2002 ▸) and semiconductors (Barchuk *et al.*, 2010 ▸) to high-*T*_c_ superconductors (Izquierdo *et al.*, 2006 ▸; Izquierdo *et al.*, 2011 ▸) and even pharmaceuticals (Chan *et al.*, 2010 ▸).

**Figure 1:**
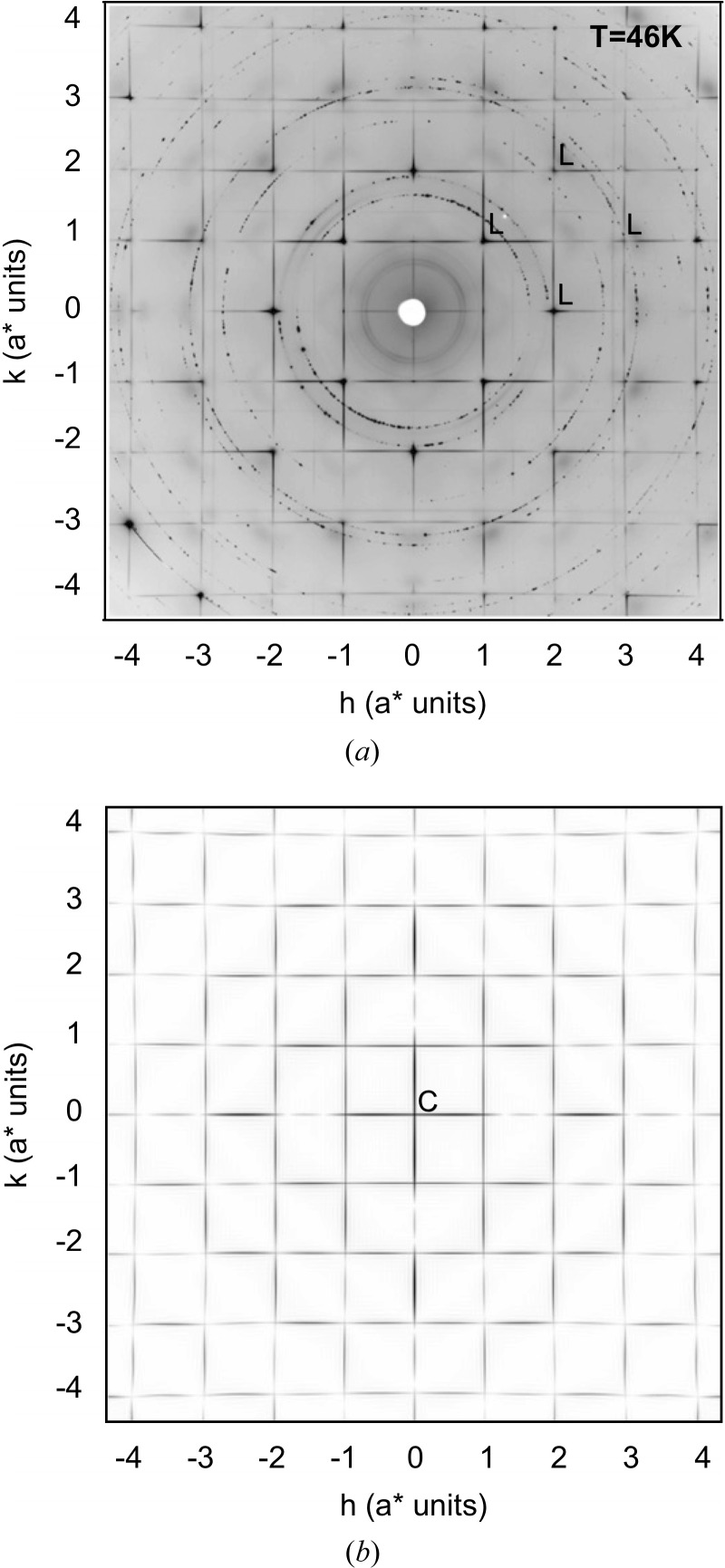
Observed and calculated *hk*0 diffraction patterns for the high-*T*_c_ superconductor HBCO. Reproduced with permission from Izquierdo *et al.* (2011 ▸), copyright (2011) Elsevier. The features marked L and C are discussed in the text. It should be noted that, because the data were recorded using a simple transmission of high-energy X-rays through a thin sample, the displayed data only approximately correspond to the (*hk*0) reciprocal plane. Near the periphery of the displayed pattern, the data derive from the first-order Laue zone and correspond to the (*hk*1) plane.

**Figure 2:**
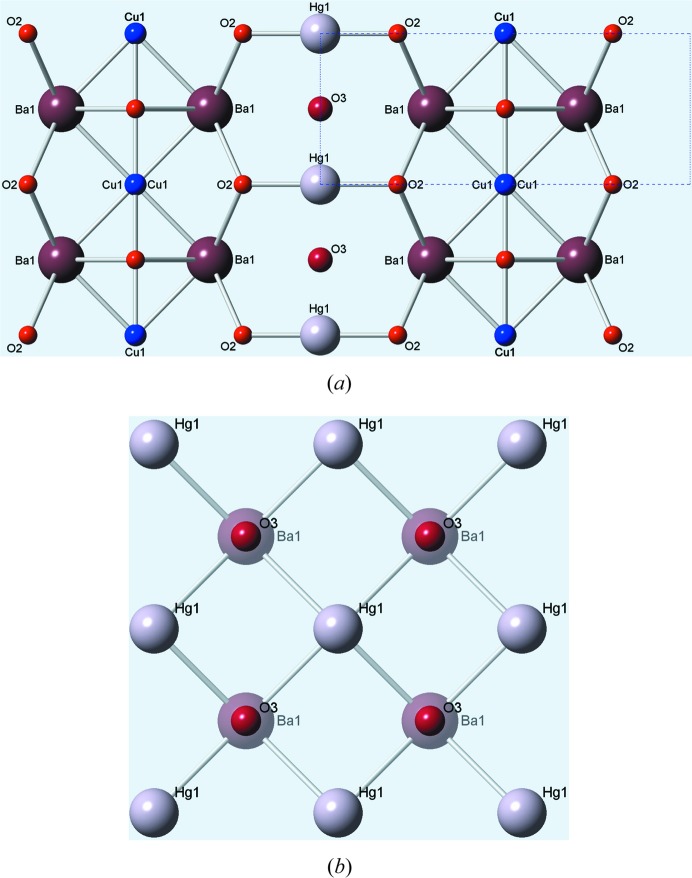
The average structure of the high-*T*_c_ superconductor HBCO, drawn using the coordinates published by Bertinotti *et al.* (1996 ▸). (*a*) A projection down [010], showing how the planar HgO_δ_ layer is sandwiched between two Ba_2_CuO_4_ layers. (*b*) The HgO_δ_ layer shown in projection down [001], showing the O3 interstitial site surrounded by four Hg atoms and sandwiched between Ba atoms in the layers below and above (not shown). The O3 site has an occupancy of δ.

To understand these materials it is not sufficient to know their average unit-cell structure as revealed by Bragg scattering. It requires additionally knowledge of their local or nanoscale structure – information that can only be obtained from the diffuse scattering component of the total scattering. Considering the above examples, it is perhaps quite surprising that, while there have been many studies of single-crystal diffuse scattering for alloys and ferroelectric materials, there have been very few for high-*T*_c_ superconductors. The reasons for this are not apparent, since the experiments reported by Izquierdo *et al.* (2011 ▸) for the high-*T*_c_ superconductor HgBa_2_CuO_4+δ_ (hereinafter referred to as HBCO) showed that very detailed single-crystal diffuse scattering (SCDS) patterns are readily obtainable using synchrotron radiation. An example section of data obtained by these authors is shown in Fig. 1 ▸(*a*) for reference. A diagram of the average structure of HBCO is shown in Fig. 2 ▸.

However, though obtaining such diffuse scattering data is now feasible for most crystalline materials, interpreting and analysing the data remain problematic. There have been some attempts to make the analysis of diffuse scattering more routine (Michels-Clark *et al.*, 2013 ▸; Simonov *et al.*, 2014 ▸) and more readily available to a wider range of researchers but, in most cases, the modelling of SCDS still relies relatively heavily on the experience of the investigator.

For the example of HBCO shown in Fig. 1 ▸(*a*), Izquierdo *et al.* developed a model to explain the diffraction patterns they had observed. In this model, the doping O3 oxygen atoms tend to segregate into long isolated one-dimensional chains in each of the two tetragonal axial directions. This ordering further induces short-range structural displacive changes that can extend up to the CuO_2_ planes. A number of different short-range models were investigated but the authors provided the calculated pattern shown in Fig. 1 ▸(*b*) as giving their ‘best fit’ to the data.

This ‘best fit’ model of Izquierdo *et al.* (2011 ▸) clearly captures some of the attributes of the observed diffraction pattern. The diffuse lines that appear in Fig. 1 ▸(*b*) result from the one-dimensional defects consisting of linear chains of interstitial O3 atoms. Since the Fourier transform of a chain (or rod) is a plane in reciprocal space, the observed diffuse lines arise from the intersection of the Ewald sphere with these planes. The width of the lines corresponds to the reciprocal of the chain length (estimated to extend over 75 unit cells on average).

However, it is clear from a comparison of Fig. 1 ▸(*b*) with Fig. 1 ▸(*a*) that the model fails to capture some key features of the diffraction:

(i) The strong cross (labelled C) that appears around the origin of the calculated pattern is virtually absent in the observed pattern. Such strong scattering at low angles is indicative of occupational disorder, whereas the virtual absence of scattering along the *h* = 0 and *k* = 0 rows in the observed pattern is indicative of displacive disorder with longitudinal correlations.

(ii) Each of the diffuse lines in the observed pattern is modulated by a function that is asymmetric across the Bragg-peak positions so that the intensity on the high-angle side is stronger than that on the low-angle side. This produces L-shaped motifs (labelled L in Fig. 1 ▸) that are characteristic of ‘size-effect’ distortions. Similar features have been observed in other systems (Matsubara & Cohen, 1985 ▸; Kreisel *et al.*, 2003 ▸).

(iii) These L-shaped motifs are seen to occur in the observed patterns only for reflections *hk*0 with *h*+*k* even. There is only a slight tendency for this behaviour in the calculated pattern.

In view of the limitations of this current model outlined above, we decided to undertake a study to try to develop a new model that agrees more convincingly with the observed data.

## 2. General considerations   

### 2.1. Scattering components   

A general expression for the intensity of diffuse scattering that allows for both short-range compositional (chemical) order and local atomic distortions can be obtained by expanding the exponential in the kinematic scattering equation in powers of displacement
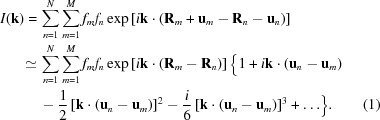
Here, *I*(**k**) is the total scattered intensity and *f_m_* is the scattering factor of atom *m* associated with the lattice site at location **R**_*m*_ and which is displaced from its site by a small amount **u**_*m*_; **k** = 2π**S** = *h*_1_ a* + *h*_2_ b* + *h*_3_ c* is the scattering vector.

Equation (1) includes both Bragg peaks and diffuse scattering. However, when the Bragg peaks are removed the diffuse part of the intensity may still be written as the sum of component intensities, each component deriving from a different power of the displacement:
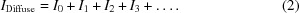
The first term *I*_0_ is independent of the displacements, the second term *I*_1_ is dependent on the first moment of displacements, the third term *I*_2_ on the second moment *etc*. *I*_0_ is commonly known as the short-range order (SRO) component, *I*_1_ as the ‘size-effect’ component, and *I*_2_ as the Huang scattering and thermal diffuse scattering (TDS) component. Expressions for each of these diffuse components may be found in Welberry & Weber (2016 ▸). The different components depend on different lattice averages, have different variations in the reciprocal space coordinates *h*_1_, *h*_2_ and *h*_3_, and also depend differently on the atomic scattering factors.

For the present purposes, it is sufficient to realise that all these different components go to zero at the origin except for the *I*_0_ SRO component. From the appearance of the observed pattern in Fig. 1 ▸(*a*), it is clear that there is only a small contribution from occupancy and the majority of the scattering comes from atomic displacements.

### 2.2. Apparent-valence or bond-valence sum method   

The apparent-valence or bond-valence sum method (Brese & O’Keeffe, 1991 ▸; Adams, 2001 ▸; Brown, 2009 ▸) is widely used in solid-state chemistry to assess the structural stability of a system. It is expected that the individual valences of the constituent atoms (or ions) in a solid will all be satisfied on a local level. Unless the apparent valences of all the atoms in a structure are close to their expected values, the structure is unlikely to be stable [see Whitfield *et al.* (2014 ▸) for a more detailed discussion]. These considerations were taken into account in developing the models for HBCO described below.

The average crystal structure determination of HBCO (Bertinotti *et al.*, 1996 ▸) places the doping O3 atoms at the (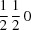) position in the unit cell at the centre of a square of four Hg ions and sandwiched between a pair of Ba ions at (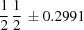) (see Fig. 2 ▸). In this position the apparent valence of the O3 atom is only 0.86, instead of its ideal value of 2.0, *i.e.* it is highly underbonded. With a value as low as 0.86 it is quite clear that the interstitial O3 atom is very uncomfortable, and significant local relaxation of the strain this entails must ensue. A number of different possible scenarios were considered for the way in which the O3 atom might achieve a valence of 2.0 and hence alleviate this strain.

### 2.3. Computer simulation   

**Figure 3:**
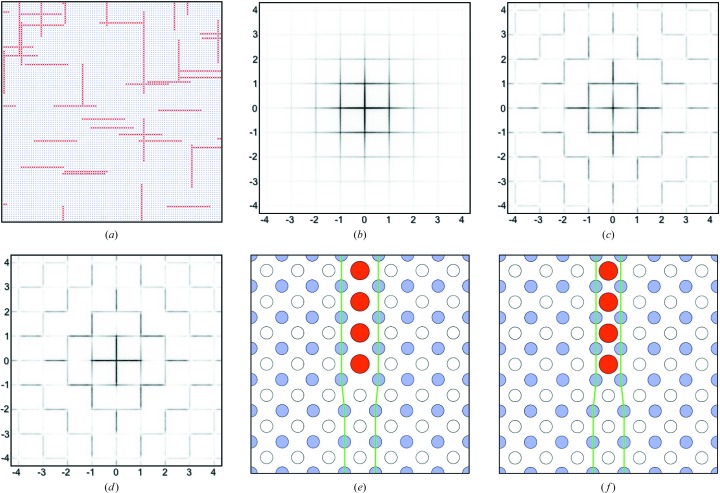
The simple linear chain SRO model. (*a*) A small region of the simulation array, showing the randomly positioned chains of interstitial O3 atoms. (*b*) The *hk*0 diffraction pattern calculated from the full (512 × 512 cells) model with all atoms on their average site positions. Note *h* is horizontal and *k* is vertical. (*c*) The *hk*0 pattern after size-effect relaxation with ξ_1_ = 0.0025. (*d*) The *hk*0 pattern after size-effect relaxation with ξ_1_ = −0.0025. (*e*) A plot showing the size-effect distortion (exaggerated ×40) around a defect corresponding to part (*c*). (*f*) A plot showing the size-effect distortion (exaggerated ×40) around a defect corresponding to part (*d*). It should be noted that the maximum intensity in part (*b*) is less than half of that in (*c*) or (*d*).

Computer simulation of a model structure has become a powerful and well accepted technique for aiding the interpretation and analysis of diffuse scattering patterns (Welberry & Butler, 1994 ▸; Welberry, 2004 ▸; Welberry & Weber, 2016 ▸). At one extreme, a very simplified model may be useful in demonstrating particular qualitative effects, while at the other a quantitative and very detailed description of a disordered structure can be obtained. In the present case, a large two-dimensional array (512 × 512) was used to represent a single layer of the structure. Initially this was used to represent a single plane of Hg ions together with the interstial O3 atoms, but subsequently the Ba atoms above and below this layer were included. It was assumed throughout that each such layer was independent of those one unit cell above or below. Fig. 3 ▸(*a*) shows a small representative region (100 × 100 unit cells) of the simulation array. The randomly positioned chains of interstitial O3 atoms are clearly seen. For the present study, the chain length was chosen for convenience to be 20 unit cells. This is less than that estimated by Izquierdo *et al.* (2011 ▸), but a shorter length allows better statistics to be obtained in a crystal of limited size. The only material difference this makes to the calculated diffraction patterns presented in the paper is that the diffuse lines are somewhat more diffuse than those in the observed data.

The diffuse diffraction patterns presented in the following examples were calculated from this array using the program *DIFFUSE* (Butler & Welberry, 1992 ▸) with a ‘lot size’ of 50 × 50 × 1 unit cells. Since the average structure is subtracted in the calculation, the resulting diffraction patterns show only the diffuse component of the scattering.

## 3. Simple chain of interstitial O3 atoms   

We first consider the simple notion that the interstitial O3 atoms form one-dimensional chains along the two axial directions. Fig. 3 ▸(*b*) shows the diffuse diffraction pattern of the *hk*0 section calculated from the full array. This is seen to consist of a series of diffuse lines for *h* = 0, ±1, ±2, ±3, ±4, … *etc.* and *k* = 0, ±1, ±2, ±3, ±4, … *etc.* Each set of lines is oriented normal to the length of the defect chain producing them. The width of the diffuse lines is reciprocally related to the length of the chain. Since there are no displacements in this simple model, the diffracted intensity seen in Fig. 3 ▸(*b*) consists solely of the SRO term *I*_0_ in equation (2) which comes only from the O3 site disorder.

For Figs. 3 ▸(*c*) and 3 ▸(*d*), local size-effect relaxation was applied to the distribution used for Fig. 3 ▸(*b*). A simple relaxation was applied in which the columns (or rows) of Hg ions neighbouring the defect chain were displaced either away from or towards the chain by an amount ξ_1_ for the nearest neighbour, ξ_2_ for the second neighbour and ξ_3_ for the third neighbour. We used a simple decay in which ξ_2_ = ξ_1_/3 and ξ_3_ = ξ_1_/9. For Fig. 3 ▸(*c*), ξ_1_ was 0.0025, and it was −0.0025 for Fig. 3 ▸(*d*). For these two patterns, the introduction of atomic displacements for Hg results in contributions to the intensity from the size-effect term *I*_1_ in equation (2) that modify the basic SRO pattern (Fig. 3 ▸ *b*). Figs. 3 ▸(*e*) and 3 ▸(*f*) show exaggerated plots of these local distortions, produced by setting ξ_1_ to ±0.1. (Note that a shift of 0.0025 corresponds to only 0.01 Å, which would not be visually discernible.)

Consideration of this simple model allows some important conclusions to be drawn. First, the actual SRO intensity from the interstial O3 atoms is very weak and is strongly modified by extremely small (∼0.01 Å) displacements of the heavy Hg atoms. Secondly, although aspects of the size-effect generated patterns (Figs. 3 ▸ *c* and 3 ▸ *d*) show some resemblance to the observed pattern of Fig. 1 ▸(*a*), two of the three key features described in Section 1 are not well reproduced. For example, while the L-shaped features on the high-angle side of 110 and 220 in Fig. 3(*d*) are similar to those in the observed pattern, the strong cross at the origin is not. There are other strong intensities on the *h* = 0 and *k* = 0 rows that are absent in the observed pattern.

## 4. Model 1   

**Figure 4:**
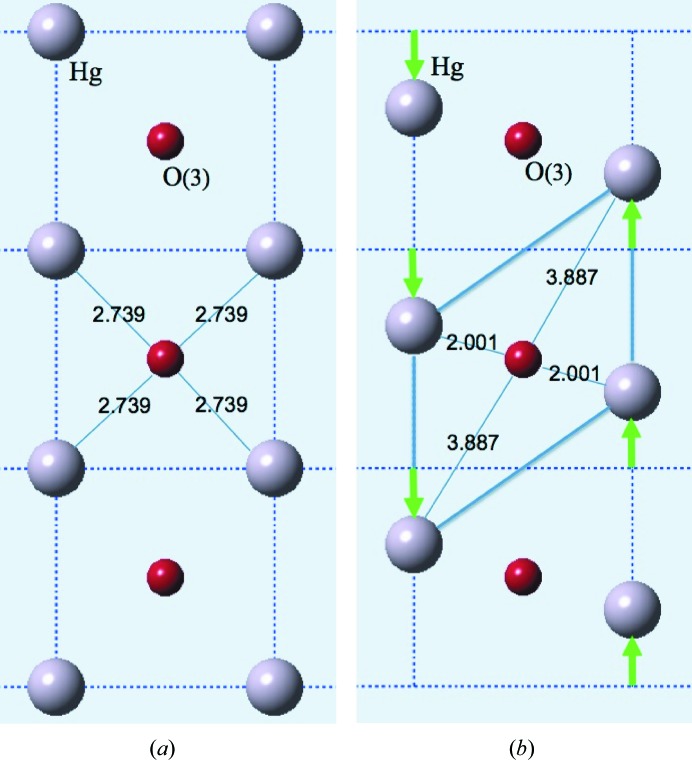
Model 1. Only the Hg ions move, by an amount δ_Hg_ = ±0.37 along *b*. (*a*) The O3 valence is 0.86. (*b*) The O3 valence is 1.98.

In the first model, we assume that the O3 atom stays at the centre of the unit-cell *ab* basal plane and the Hg atoms at the corners shift along the chain direction in opposite directions by an amount ±δ_Hg_ to produce a sheared cell (see Fig. 4 ▸). This results in two short and two long Hg—O bonds. Table 1 ▸ gives values of the contributions to the valence sum from the six contributing cations, four Hg plus two Ba, as δ_Hg_ is varied. Note that, in order to obtain a valence close to 2.0, δ_Hg_ must be ∼0.37. At this point the O3 atom is essentially only bonded to two of the four Hg atoms and the two out-of-plane Ba atoms. Note also that, even when δ_Hg_ is as much as 0.2, the O3 atom is still very underbonded.

**Figure 5:**
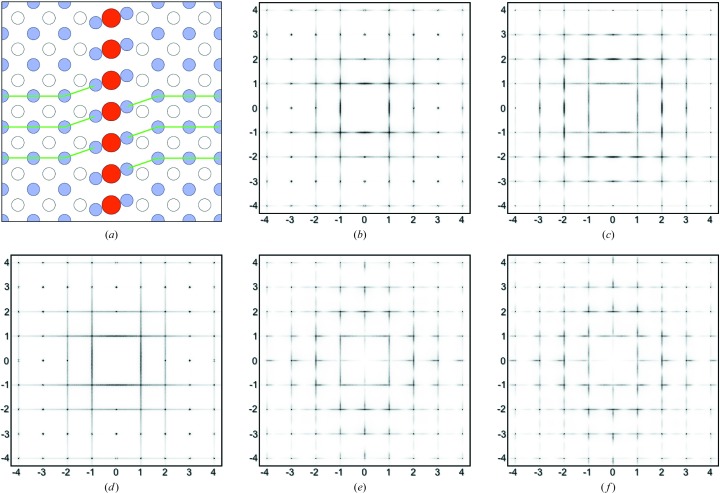
Model 1 simulation results. (*a*) The region in the simulation array showing a chain of interstitial O3 atoms (red) with displaced neighbouring columns of Hg ions (blue). Open circles are vacant O3 sites. Parts (*b*) to (*f*) are *hk*0 diffraction patterns calculated with different values of the Hg shift δ_Hg_ and the size-effect parameter ξ_1_. (*b*) δ_Hg_ = 0.35, ξ_1_ = 0.0. (*c*) δ_Hg_ = 0.2, ξ_1_ = 0.0. (*d*) The same as part (*b*) but with only a single column of displaced Hg atoms. (*e*) δ_Hg_ = 0.35, ξ_1_ = −0.1. (*f*) δ_Hg_ = 0.35, ξ_1_ = 0.1.

To calculate the diffraction pattern of this model, the same basic distribution of defects as shown in Fig. 3 ▸(*a*) was used. Now the defect comprises the linear chains of O3 atoms but, in addition, the neighbouring rows or columns of Hg ions are displaced by amounts ±δ_Hg_, as indicated in Fig. 4 ▸(*b*). Figs. 5 ▸(*b*) and 5 ▸(*c*) show *hk*0 diffraction patterns corresponding to two different values of δ_Hg_. These show how the value of δ_Hg_ influences the intensity in the different orders of diffuse line. Note that the modulation of intensity along the diffuse lines arises because the structure factor of the defect involves a double column (or row) of Hg sites. For comparison, we show in Fig. 5 ▸(*d*) a pattern calculated with the δ_Hg_ displacement in one of the two columns set to zero. With displacements now only in a single column, each diffuse line has a smooth unmodulated intensity profile.

It should also be noted that, with these magnitudes of shift for the strongly scattering Hg ions, any contribution from the SRO occupancy term *I*_0_ for the much more weakly scattering O3 atoms is not discernible on this scale.

### 4.1. Size-effect relaxation   

The displacement δ_Hg_ used in this section to define the particular linear defect (Model 1) is a fairly major displacement corresponding to shifts of the Hg ions from their sites in the average crystal structure by ∼1.4 Å. However, since the number of interstitial O3 atoms is small (∼6%), the effect on the overall average lattice is still relatively small. These linear defects thus represent rather major, though infrequently occurring, interuptions to the crystal lattice periodicity.

However, in addition to these primary displacements, it is to be expected that there will be additional size-effect distortions in the form of lateral atomic shifts, either towards or away from the defect, in order to alleviate local strain. These lateral shifts will produce significant redistribution of the diffracted intensity, as described by the *I*_2_ term in equation (2). The same simple relaxation scheme used in Section 3 was applied to the example of Fig. 5 ▸(*b*). The columns (or rows) of Hg ions neighbouring the defect chain were displaced (away from or towards) the defect chain by an amount ±ξ_1_ for the nearest-neighbour, ±ξ_2_ for the second-neighbour and ±ξ_3_ for the third-neighbour columns. As before, we assumed a simple decay in which ξ_2_ = ξ_1_/3 and ξ_3_ = ξ_1_/9.

In contrast with the example in Section 3, the values of ξ_1_ required here are much higher. For the diffraction patterns in Figs. 5 ▸(*e*) and 5 ▸(*f*), values of ξ_1_ = −0.1 and ξ_1_ = 0.1 were used, respectively. Note that a positive value corresponds to movement away from the defect. It is seen from these two figures that the size-effect relaxation has redistributed the intensity in the diffuse streaks and L-shaped motifs have resulted. In Fig. 5 ▸(*f*), the intensity is stronger on the high-angle side of the Bragg positions, while in Fig. 5 ▸(*e*) the intensity is stronger on the low-angle side. Although the intensity transfer in Fig. 5 ▸(*f*) is in the same direction as in the observed pattern, there are two major points of disagreement. First, the L-shaped motifs only extend halfway along the reciprocal cell edges, whereas in the observed pattern they extend essentially from one Bragg position to the next. This clearly arises because of the structure-factor modulation due to the double column of displacements seen in Fig. 5 ▸(*b*). Secondly, the L-shaped motifs are not confined to *h* + *k* = even reflections.

These observations led to the conclusion that the primary displacement should only involve a single column/row of ions. One possibility might be to use the displacement of a single column of Hg ions, as in Fig. 5 ▸(*d*), but this idea was discarded since the defect created is unsymmetric and difficult to justify from a physical point of view. Consequently, Model 2 (see below) was developed, in which the column of ions involved in the primary displacement is that involving the Ba_2_O units that occur between two Hg columns.

## 5. Model 2   

**Figure 6:**
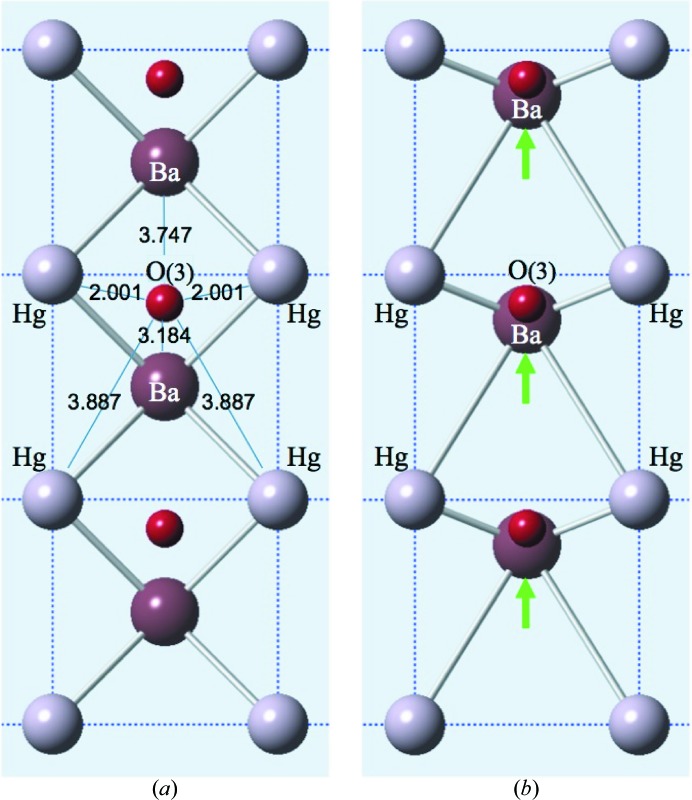
Model 2. Hg ions do not move. (*a*) O3 moves δ = ±0.37 along *b*. (*b*) O3 moves δ = ±0.37 and Ba moves δ = ±0.3. The O3 valence in (*a*) is 1.749 and in (*b*) is 1.965.

We assume that the Hg ions remain on (or close to) their average sites at the corners of the unit cell and that the O3 atoms try to achieve their required valency of 2.0 by shifting along *b* closer to two of the four Hg atoms (see Fig. 6 ▸ *a*). If the Ba ions above and below the plane containing the Hg and O3 atoms remain on their average sites at (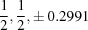), then the Ba—O3 bonds are somewhat lengthened and this diminishes the apparent valence of the O3 atoms. Nevertheless, the O3 apparent valence is now 1.749, considerably better than in the unperturbed average lattice (see Table 2 ▸). However, even this apparent valence is sufficiently far from 2.0 to provide an incentive for further ionic shifts to reduce the strain. In Model 2 we assume that this is achieved by the Ba ions undergoing a shift δ_Ba_ along *b* to restore their proximity to the displaced O3 atoms. Table 2 ▸ gives values of the apparent valence of O3 when δ_Ba_ = 0.1 and δ_Ba_ = 0.3. In the latter case the valence is close to the desired value of 2.0, and this arrangement (shown in Fig. 6 ▸ *b*) was adopted for use in simulations of this model.

**Figure 7:**
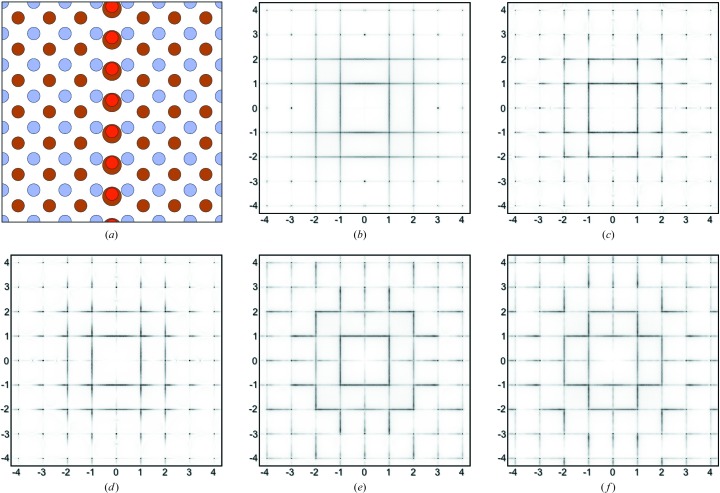
Model 2 simulation results. (*a*) A region in the simulation array showing a chain comprising displaced O3 atoms (red) and displaced Ba ions (brown). Blue circles are Hg ions. Parts (*b*) to (*f*) are *hk*0 diffraction patterns calculated with different values of the Ba shift δ_Ba_ and the size-effect shifts ξ_Ba_ and ξ_Hg_. (*b*) δ_Ba_ = 0.3, ξ_Ba_ = ξ_Hg_ = 0. (*c*) δ_Ba_ = 0.3, ξ_Ba_ = −0.04. (*d*) δ_Ba_ = 0.3, ξ_Ba_ = 0.04. (*e*) δ_Ba_ = 0.3, ξ_Hg_ = −0.04. (*f*) δ_Ba_ = 0.3, ξ_Hg_ = 0.04.

Fig. 7 ▸(*a*) shows schematically the arrangement of ions around a defect for this system. The red O3 atoms are shown to identify the defect but, as for the previous example, they contribute very little to the scattering compared with the displacement scattering of the heavy metal ions (Ba in this case). The brown circles represent Ba ions and the blue circles the Hg ions. The displaced Ba ions are drawn rather larger than the undisplaced ones in order to be able to see them behind the O atoms. Fig. 7 ▸(*b*) shows the corresponding diffraction pattern obtained for a Ba displacement of δ_Ba_ = 0.3, with all other ions fixed to their average site positions. The scattering in this picture comes almost entirely from the Ba atoms. As for the case of Model 1, varying the magnitude of this shift affects the relative magnitude of the different orders of diffuse line.

### 5.1. Size-effect relaxation   

Two different schemes for applying local size-effect relaxation around the defects were investigated.

(i) In Scheme 1 it was assumed that relaxation occurs in the Ba layers only. The same kind of relaxation was carried out as for previous models. Neighbouring columns of Ba ions were displaced laterally either towards or away from the defect column by an amount ±ξ_1_. In this case it was found that a value of ξ_1_ = 0.04 gave satisfactory results. As before, progressively diminishing values for ξ_2_ and ξ_3_ were also used. For this relaxation scheme the Hg ions remained on their average site positions.

(ii) In Scheme 2 it was assumed that the relaxation occurs in the Hg layers only. After the initial primary displacement of the Ba ions by a shift δ_Ba_, subsequent size-effect shifts, ξ_1_, ξ_2_ and ξ_3_, either away from or towards the defect chains were carried out on the neighbouring columns of Hg ions. Values of ξ_1_ = ± 0.04 were used in this case too.

The resulting effects on the *hk*0 diffraction patterns are shown in Figs. 7 ▸(*c*) and 7 ▸(*d*) for Scheme 1 and in Figs. 7 ▸(*e*) and 7 ▸(*f*) for Scheme 2. In both cases, the size-effect relaxation causes a transfer of intensity from one side of the Bragg position to the other to produce the L-shaped motifs. For ξ_1_ < 0.0, the transfer is from high angle to low, while for ξ_1_ > 0.0 the transfer is from low angle to high. It is this latter direction of transfer that corresponds to that observed in the X-ray data (Fig. 1 ▸ *a*). This corresponds to the neighbouring layers shifting away from the defect.

There are two main differences between the patterns in Figs. 7 ▸(*c*) and 7 ▸(*d*) and those in Figs. 7 ▸(*e*) and 7 ▸(*f*). Firstly, it is apparent in Figs. 7 ▸(*c*) and 7 ▸(*d*) that the arms of the L-shaped motifs only extend about halfway along the vectors to the next Bragg positions. By contrast, the arms of the L-shaped motifs in Figs. 7 ▸(*e*) and 7 ▸(*f*) extend virtually the whole way to the next Bragg position. Secondly, it is quite clear that in Figs. 7 ▸(*c*) and 7 ▸(*d*) the motifs are associated with all Bragg peaks, including those with *h* + *k* = even and *h* + *k* = odd. On the other hand, in Figs. 7 ▸(*e*) and 7 ▸(*f*) the motifs are only associated with *h* + *k* = even peaks.

## 6. Final model comparison   

**Figure 8:**
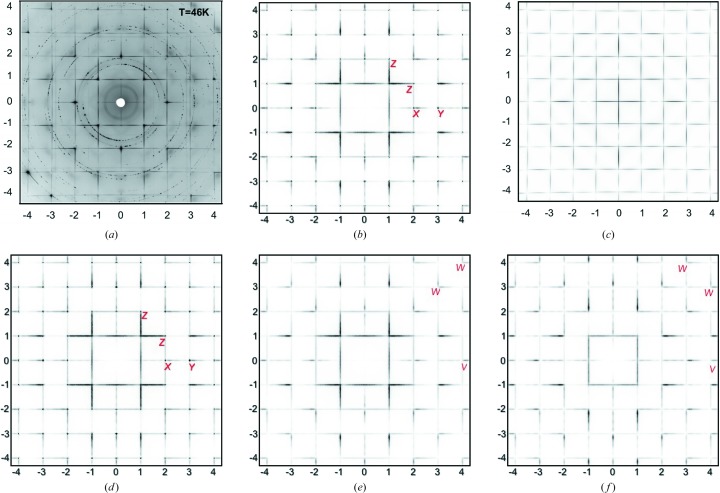
Comparison of results for the final model of the present work with the work of Izquierdo *et al.* (2011 ▸). (*a*) Observed X-ray data and (*c*) Izquierdo’s ‘best-fit’ model [both reproduced with permission from Izquierdo *et al.* (2011 ▸), copyright (2011) Elsevier]. (*b*) The *hk*0 diffraction pattern calculated using δ_Ba_ = 0.40, ξ_Hg_ = 0.04 and ξ_Ba_ = 0.02. (*d*) The same as part (*b*) but with the ξ_Ba_ term omitted. Parts (*e*) and (*f*) show, respectively, (*hk* 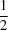) and (*hk*1.0) patterns calculated for the same model as part (*b*).

In the light of the two different relaxation modes described in Section 5.1, close inspection of the observed diffraction pattern suggested that the main relaxation producing the size-effect intensity transfer involves the neighbouring Hg columns (rows). However, the arms of the L-shaped feature near 110 in the observed data (reproduced in Fig. 8 ▸ *a* for convenience) do seem to diminish in intensity as the 210 or 120 peaks are approached, and this requires some involvement of the nearest-neighbour Ba columns as well as the Hg columns. Consequently, we show in Fig. 8 ▸(*b*) the calculated diffraction pattern of a model with δ_Ba_ = 0.40, ξ_Hg_ = 0.04 and ξ_Ba_ = 0.02. For comparison, Fig. 8 ▸(*d*) shows the same model but with the ξ_Ba_ term omitted. Note the difference in intensity at the points marked *Z*. Including the ξ_Ba_ term in Fig. 8 ▸(*b*) also has the beneficial effect of reducing the intensity of the small streak at *Y*, where it is much weaker than the same peak in Fig. 8 ▸(*d*). In addition, the streak at *X* is more prominent in Fig. 8 ▸(*b*), in better agreement with the observed data.

Both patterns (Figs. 8 ▸ *b* and 8 ▸ *d*) show similar peaks near (400) and (040) that are not seen in the observed pattern. However, it should be remembered that, as mentioned earlier (see Fig. 1 ▸), near its periphery the observed pattern is derived from data closer to the (*hk*1) plane rather than the (*hk*0) plane. The same reason may be invoked to explain why the systematic appearance of the L-shaped motifs near only *h* + *k* = even reflections is broken for reflections such as (340) and (430) near the periphery of the pattern.

This has been confirmed by computing diffraction patterns for (*hk* 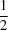) and (*hk*1.0) (shown in Figs. 8 ▸ *e* and 8 ▸ *f*, respectively). While for (*hk* 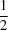) the pattern is similar to Fig. 8 ▸(*b*), for (*hk*1.0) the L-shaped features marked *W* occur for *h* + *k* 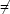 even. Although the peaks near (401) (marked *V*) and (041) are present in Fig. 8 ▸(*f*), the overall intensity in this section is considerably weaker than the corresponding (*hk*0.0) section, and hence these streaks would appear less prominent in a composite pattern.

In conclusion, we consider that Fig. 8 ▸(*b*) satisfactorily addresses the points of discrepancy of the Izquierdo model outined in Section 1 and is considered the ‘best fit’ to the observed data that has been obtained in the current study.

## 7. Discussion   

The model of Fig. 8 ▸(*b*) is very simple, comprising as it does only four adjustable parameters: δ_Ba_, the magnitude of the Ba displacement from its average position; ξ_Hg_, the induced lateral shift of the nearest-neighbour columns of Hg ions; ξ_Ba_, the induced lateral shift of the nearest-neighbour columns of Ba ions; and δ_O3_, the magnitude of the O3 displacement from its average position. This last parameter has little direct effect on the diffraction pattern, since the scattering from the oxygen is very weak compared with the displacement scattering of the heavy ions. It is, however, the key to understanding the defect structure, since it is the main driving force that enables the O3 oxygen to achieve an apparent valence close to 2.0.

The correlated displacements of the Ba ions associated with the defect chain of O3 ions is the prime source of the so-called ‘transverse-polarized’ set of diffuse planes that are seen in Fig. 7 ▸(*b*) and in subsequent patterns presented in Figs. 7 ▸ and 8 ▸. This term ‘transverse polarized’ is commonly used in a modulation wave description of disorder and corresponds to situations where modulation wavevectors are transverse to the atomic displacements. In a correlation description of disorder (as used here), the correlations are longitudinal correlations, since the direction of the correlation is parallel to the displace­ment direction. In either description, the net result is that the diffuse plane going through the origin has zero intensity (*i.e.* in this case the lines *h* = 0 and *k* = 0).

Subsequent size-effect relaxation results in this perfect transverse polarization being broken to some extent as non-transverse-polarized modulations are induced. In the observed data (Fig. 1 ▸ *a*), there is evidence for this in the quite strong streak that occurs on the high-angle side of the 200 and 020 reflections. Although the final calculated pattern in Fig. 8 ▸(*b*) shows small differences from the observed data, these discrepancies may largely be attributed to the fact that the observed pattern is not truly a plane (*hk*0) section but tends towards (*hk*1) near the periphery.

Despite its simplicity, this final model produces a diffraction pattern that resembles the observed data much more closely than the model originally reported by Izquierdo *et al.* (2011 ▸). In particular, this new model addresses the three key aspects of the inadequacy of the original fit outlined in Section 1. The new model has also taken into account the apparent valence requirements of the constituent atoms in the disordered layer, and the magnitude of the displacements δ_Ba_ and δ_O3_ suggested by such calculations are in good agreement with the values deduced from the diffraction alone.

While the present results are very encouraging, there are still some issues that future work needs to resolve. It is likely that the large Ba shifts would have a carry-on effect to the CuO_2_ layer beneath and produce displacements in this layer that would further affect the details of the diffraction pattern. No attempt has been made at present to involve atoms in this layer, though in the context of understanding the superconductivity this is clearly important. Before such an attempt is made, it would be beneficial to obtain more extensive (three-dimensional) diffuse scattering data. Such data would also allow assessment of out-of-plane displacements and how these might contribute to the apparent valence-sum calculations.

In addition to the diffuse streaks that have been discussed in this paper, the observed data also contain broader regions of weaker scattering. In many instances these occur beneath the L-shaped motifs. These broad spots were attributed by Izquierdo *et al.* (2011 ▸) to acoustic phonons. Such features correspond to the *I*_2_ Huang scattering and thermal diffuse scattering (TDS) component in equation (2). No attempt has been made to model this in the present study. Such scattering has been modelled in numerous previous studies using Monte Carlo simulation of a harmonic spring model of the disordered structure. Development of such a model could also be fruitful in terms of assessing the apparent valence of all the atoms in the structure using, for instance, the GII (global instability index) [see, for example, Whitfield *et al.* (2014 ▸)].

A second feature of the observed pattern that we have not attempted to address in this paper are the streaks that occur at incommensurate values of *h* and *k*. The strongest of these streaks may be seen at *h*, *k* ≃ 2.8, with a weaker one at *h*, *k* ≃ 1.4. Although much weaker than the streaks accounted for by the present model, they are similar in appearance and are similarly transverse polarized. Their observation seems to indicate the presence of a second phase with a smaller unit-cell dimension of ∼2.76 Å.

## Figures and Tables

**Table 1:** Values of the apparent valence, *V_i_* = Σ_*j*_ν_*ij*_, for the O3 atom in Model 1 *d_j_* are the distances to the coordinating cations and the numbers in parentheses are the individual contributions, ν_*ij*_, to the valence sum for each cation *j*.

δ_Hg_	*V_i_*	*d*_Hg1_	*d*_Hg2_	*d*_Hg3_	*d*_Hg4_	*d*_Ba_
0.0	0.86	2.739	2.739	2.739	2.739	2.843
		(0.109)	(0.109)	(0.109)	(0.109)	(0.224†)
0.20	1.25	2.259	3.333	2.259	3.333	2.843
		(0.38)	(0.02)	(0.38)	(0.02)	(0.224†)
0.37	1.98	2.001	3.887	2.001	3.887	2.843
		(0.76)	(0.01)	(0.76)	(0.01)	(0.224†)

† Value ×2 because there are two Ba neighbours.

**Table 2:** Values of the apparent valence, *V_i_* = Σ_*j*_ν_*ij*_, for the O3 atom in Model 2 *d_j_* are the distances to the coordinating cations and the numbers in parentheses are the individual contributions, ν_*ij*_, to the valence sum for each cation *j*.

δ_Ba_	*V_i_*	*d*_Hg1,2_	*d*_Hg3,4_	*d*_Ba1_	*d*_Ba2_ †
0.0	1.749	2.001	3.887	3.184	3.747
		(0.761‡)	(0.005‡)	(0.089‡)	(0.019‡)
0.10	1.803	2.001	3.887	3.029	4.010
		(0.761‡)	(0.005‡)	(0.136‡)	(0.010‡)
0.30	1.965	2.001	3.887	2.856	4.589
		(0.761‡)	(0.005‡)	(0.217‡)	(0.002‡)
0.375	1.999	1.997	3.904	2.856	4.769
		(0.769‡)	(0.004‡)	(0.224‡)	(0.001‡)

† Ba2 is in the next unit cell along *b* from Ba1 (see Fig. 6 ▸).

‡ Value ×2 because there are two of each vector length.

## References

[bb1] Adams, St. (2001). *Acta Cryst.* B**57**, 278–287. 10.1107/s0108768101003068 11373385

[bb2] Barchuk, M., Holý, V., Miljević, B., Krause, B., Baumbach, T., Hertkorn, J. & Scholz, F. (2010). *J. Appl. Phys.* **108**, 043521.

[bb3] Bertinotti, A., Viallet, V., Colson, D., Marucco, J., Hammann, J., Le Bras, G. & Forget, A. (1996). *Phys. C Supercond.*, **268**, 257–265.

[bb4] Brese, N. E. & O’Keeffe, M. (1991). *Acta Cryst.* B**47**, 192–197.

[bb5] Brown, I. D. (2009). *Chem. Rev.* **109**, 6858–6919. 10.1021/cr900053k PMC2791485 19728716

[bb6] Butler, B. D. & Welberry, T. R. (1992). *J. Appl. Cryst.* **25**, 391–399.

[bb7] Chan, E. J., Welberry, T. R., Heerdegen, A. P. & Goossens, D. J. (2010). *Acta Cryst.* B**66**, 696–707. 10.1107/S0108768110037055 PMC2992034 21099031

[bb8] Izquierdo, M., Megtert, S., Albouy, J. P., Avila, J., Valbuena, M. A., Gu, G., Abell, J. S., Yang, G., Asensio, M. C. & Comes, R. (2006). *Phys. Rev. B*, **74**, 054512.

[bb9] Izquierdo, M., Megtert, S., Colson, D., Honkimäki, V., Forget, A., Raffy, H. & Comès, R. (2011). *J. Phys. Chem. Solids*, **72**, 545–548.

[bb10] Keen, D. A. (2002). *J. Phys. Condens. Matter*, **14**, R819–R857.

[bb11] Kreisel, J., Bouvier, P., Dkhil, B., Thomas, P. A., Glazer, A. M., Welberry, T. R., Chaabane, B. & Mezouar, M. (2003). *Phys. Rev. B*, **68**, 014113.

[bb12] Matsubara, E. & Cohen, J. B. (1985). *Acta Metall.*, **33**, 1945–1955.

[bb13] Michels-Clark, T. M., Lynch, V. E., Hoffmann, C. M., Hauser, J., Weber, T., Harrison, R. & Bürgi, H. B. (2013). *J. Appl. Cryst.* **46**, 1616–1625.

[bb14] Paściak, M., Welberry, T. R., Kulda, J., Kempa, M. & Hlinka, J. (2012). *Phys. Rev. B*, **85**, 224109.

[bb15] Schweika, W. (1998). *Disordered Alloys: Diffuse Scattering and Monte Carlo Simulations.* Heidelberg: Springer.

[bb16] Simonov, A., Weber, T. & Steurer, W. (2014). *J. Appl. Cryst.* **47**, 1146–1152.

[bb17] Welberry, T. R. (2004). *Diffuse X-ray Scattering and Models of Disorder.* Oxford University Press.

[bb18] Welberry, T. R. & Butler, B. D. (1994). *J. Appl. Cryst.* **27**, 205–231.

[bb19] Welberry, T. R. & Paściak, M. (2011). *Metall. Mater. Trans. A*, **42**, 6–13.

[bb20] Welberry, T. R. & Weber, T. (2016). *Crystallogr. Rev.* **22**, 2–78.

[bb21] Whitfield, R. E., Welberry, T. R., Paściak, M. & Goossens, D. J. (2014). *Acta Cryst.* A**70**, 626–635.

[bb22] Yamamoto, T., Choi, M.-S., Majima, S., Fukuda, T. & Kakeshita, T. (2008). *European Phys. J. Spec. Top.* **158**, 1–5.

